# The impact of autotrophic versus heterotrophic nutritional pathways on colony health and wound recovery in corals

**DOI:** 10.1002/ece3.4531

**Published:** 2018-10-18

**Authors:** Elizabeth M. Burmester, Adrienne Breef‐Pilz, Nicholas F. Lawrence, Les Kaufman, John R. Finnerty, Randi D. Rotjan

**Affiliations:** ^1^ Billion Oyster Project New York New York; ^2^ Department of Biology Boston University Boston Massachusetts; ^3^ John H Prescott Marine Laboratory Anderson‐Cabot Center for Ocean Life, New England Aquarium Boston Massachusetts

**Keywords:** coral, nutrition, recovery, symbiosis

## Abstract

For animals that harbor photosynthetic symbionts within their tissues, such as corals, the different relative contributions of autotrophy versus heterotrophy to organismal energetic requirements have direct impacts on fitness. This is especially true for facultatively symbiotic corals, where the balance between host‐caught and symbiont‐produced energy can be altered substantially to meet the variable demands of a shifting environment. In this study, we utilized a temperate coral–algal system (the northern star coral, *Astrangia poculata,* and its photosynthetic endosymbiont, *Symbiodinium psygmophilum*) to explore the impacts of nutritional sourcing on the host's health and ability to regenerate experimentally excised polyps. For fed and starved colonies, wound healing and total colony tissue cover were differentially impacted by heterotrophy versus autotrophy. There was an additive impact of positive nutritional and symbiotic states on a coral's ability to initiate healing, but a greater influence of symbiont state on the recovery of lost tissue at the lesion site and complete polyp regeneration. On the other hand, regardless of symbiont state, fed corals maintained a higher overall colony tissue cover, which also enabled more active host behavior (polyp extension) and endosymbiont behavior (photosynthetic ability of *Symbiondinium*). Overall, we determined that the impact of nutritional state and symbiotic state varied between biological functions, suggesting a diversity in energetic sourcing for each of these processes.

## 1 INTRODUCTION

The differential utilization of alternative energy sources can directly influence an organism's growth, reproduction, behavior, and survival (Heino & Kaitala, 2001). In organisms that can obtain carbon flexibly from multiple pathways, energetic dynamics can be particularly complex. For example, corals harboring photosynthetic algal symbionts (*Symbiodinium*) can obtain energy through transfer of photosynthate from the endosymbiont or by predation on plankton (Grottoli, Rodrigues, & Palardy, 2006; Palardy et al., 2008). When obtaining energy via photosynthesis, the coral holobiont (host animal plus symbionts) is functioning as an autotroph, and when obtaining its energy via predation, it is functioning as a heterotroph. However, corals often obtain energy through multiple sources simultaneously, and there may be interactions between autotrophy and heterotrophy. Additionally, as colonial organisms, energy must be translocated across individual polyps to maintain colony function (Fine, Oren, & Loya, 2002; Oren, Rinkevich, & Loya, 1997). As such, energy budgeting within coral colonies is complex, dynamic, and not yet well understood.

For tropical corals in well‐lit, shallow environments, host colonies can meet or exceed their metabolic needs through transfer of photosynthate from *Symbiodinium* spp. (Muscatine, 1990). It has been hypothesized that these corals prey on zooplankton mainly to supplement the energy they receive from the endosymbiont and to supply essential nutrients (such as phosphorus and nitrogen; Johannes, Cole, & Kuenzel, 1970; Tanaka, Miyajima, Koike, Hayashibara, & Ogawa, 2006) and that prolonged heterotrophic compensation may be a stress response that increases resilience under conditions unfavorable to autotrophy (Hughes & Grottoli, 2013; Levas et al., 2015). Additionally, heterotrophic feeding can enhance growth rate, protein, and chlorophyll concentrations, as well as calcification rates in daylight and in darkness (Ferrier‐Pagès, Witting, Tambutté, & Sebens, 2003; Houlbrèque, Tambutté, & Ferrier‐Pagès, 2003). However, the degree to which a colony can supplement lost photosynthetic resources appears to vary by species (Anthony & Fabricius, 2000; Grottoli et al., 2006), and studies have suggested that the balance between energy sources might not be fixed (Piniak, 2002).

In the temperate realm, a highly variable environment can lead to a wide variety of flexible feeding strategies, such as those employed by facultatively symbiotic corals like *Astrangia poculata *(= *A. danae*; Peters, Cairns, Pilson, & Wells, 1988, Figure 1), *Oculina patagonica* (Fine, Zibrowius, & Loya, 2001), and *Oculina arbuscula* (Leal et al., 2014). Heterotrophy has many effects on the metabolism and physiology of these facultatively symbiotic temperate corals: (a) It can mitigate thermally induced “bleaching” (a sharp reduction in symbiont density caused by exposure to elevated temperatures; Aichelman et al., 2016); (b) it increases nitrogen uptake and excretion (Szmant‐Froelich & Pilson, 1984); (c) it increases calcification and growth (Jacques & Pilson, 1980; Jacques, Marshall, & Pilson, 1983; Miller, 1995); and (d) it reduces damage from sedimentation (Peters & Pilson, 1985). Symbiotic state can impact the effects of heterotrophy, although the presence of photosynthetic symbionts does not preclude heterotrophy. For example, symbiotic colonies of *A. poculata* can retain more carbon (^14^C) from heterotrophic sources than aposymbiotic colonies (Szmant‐Froelich, 1981), and there is evidence for transfer of photosynthetic carbon to coral host tissue (Schiller, 1993). Additionally, the endosymbiont in fed *A. poculata* colonies fix carbon more efficiently (but translocate less ^14^C) than their starved counterparts (Szmant‐Froelich, 1981). This suggests a potentially high degree of interconnectivity between energy strategies (Piniak, 2002) as well as a complex dynamic between simultaneous autotrophy and heterotrophy.

**Figure 1:**
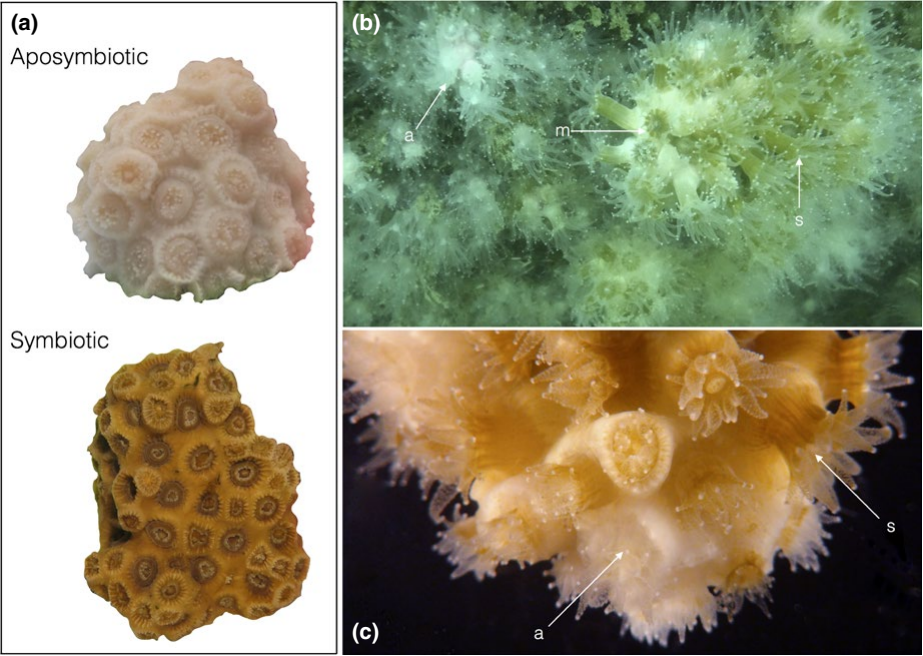
(a) Symbiont states in *Astrangia poculata*: With polyps contracted, fully aposymbiotic colonies appear white in color; fully symbiotic colonies appear brown in color. (b) [a] Aposymbiotic, [m] mottled, and [s] symbiotic colonies of *A. poculata* occur concurrently in the field. (c) Adjacent polyps in a mottled colony demonstrate [a] aposymbiotic and [s] symbiotic densities. Photographs by E.M. Burmester

The northern star coral *A. poculata* has an expansive range along the east coast of North America, from Florida and the Gulf of Mexico to southern Massachusetts (Dimond & Carrington, 2007; Dimond et al., 2013). In nature, these corals can exist in one of three basic symbiotic states with the endosymbiont, *Symbiodinium psygmophilum* (Lajeunesse, Parkinson, & Reimer, 2012): Fully symbiotic corals appear brown; aposymbiotic corals harbor far fewer symbionts, and they appear white; symbiont density can also vary from polyp to polyp, producing a mottled, mixed colony comprising both white and brown polyps (Cummings, 1983). Unlike in tropical corals, in *A. poculata*, the aposymbiotic state is not the result of stress (i.e., bleaching); white colonies of *A. poculata* are as “healthy” as brown colonies and can persist indefinitely in nature (Grace, 2004). The relatively low density of *S. psygmophilum* is actively maintained by the regular expulsion of the symbiont (Dimond & Carrington, 2008). Regardless of symbiont state, temperate colonies rely heavily on heterotrophy (Farrant, Borowitzka, Hinde, & King, 1987; Szmant‐Froelich & Pilson, 1984), with symbiont density only explaining an estimated 23% of growth in the field (Dimond & Carrington, 2007).

This study investigates the interaction of feeding and symbiotic state on wound healing in *Astrangia poculata*. There is ample evidence to suggest that both lesion recovery and colony maintenance (i.e., maintaining a healthy layer of tissue cover) are energetically costly activities that are often in conflict with each other and in conflict with other critical physiological functions such as reproduction, calcification, and growth (Anthony, Connolly, & Willis, 2002; Richmond, 1987; Rinkevich, 1996; Rotjan and Lewis, 2009; Ward, 1995). In addition, the process of lesion repair can require a high degree of colonial energy integration, which can vary by wound and colony characteristics (Oren, Benayahu, Lubinevsky, & Loya, 2001; Szmant‐Froelich, Yevich, & Pilson, 1980).

Because of its flexibility and tractability, *A. poculata* makes an ideal study organism for investigating the dynamics between energy sourcing and organismal health. This study uses (a) small‐scale wound lesion and total colony tissue recovery, (b) foraging behavior, and (c) symbiont density and photosynthetic efficiency metrics to assess colony health and stress response in the presence and absence of both autotrophic and heterotrophic nutritional strategies in naturally occurring symbiotic and aposymbiotic *A. poculata* colonies.

## 2 MATERIALS AND METHODS

### 2.1 Collection and husbandry

Colonies of *Astrangia poculata* in both symbiotic or aposymbiotic states were collected between 6 and 10 m depths at Fort Wetherill State Park in Jamestown RI (41 28′40″N, 71 21′34″W) in Summer 2014. Specimens were housed at the New England Aquarium and provided lighting in 10‐hr light cycles (14 hr dark) via T5 HO fluorescent lighting fixtures (Hamilton Technology, Gardena, CA, Aruba Sun T5‐V Series) as well as filtered, UV‐treated seawater from the Boston Harbor. Light levels (PAR) and water quality (pH, nitrate, ammonia, alkalinity) were measured weekly to ensure consistent water quality parameters. All experimental colonies were acclimated at 18°C for at least two weeks before the start of experimentation. During this acclimation period, colonies were given individualized, daily feedings of a frozen copepod slurry (50 g copepod/l; JEHM Co., Inc.).

### 2.2 Experimental setup and nutrition manipulation

Symbiotic and aposymbiotic colonies were paired by approximate size (mass), and these pairs were subsequently sorted randomly into one of four treatment groups: (a) fed/wounded, (b) fed/un‐wounded [control], (c) starved/wounded, (d) starved/un‐wounded. No significant differences were found between groups in colony mass (ANOVA, *F*_(3,220)_ = 1.3864, *p* = 0.2478; average mass −6.34 g (±0.25 *SEM*)). Overall, 28 paired symbiotic and aposymbiotic colonies were included in each treatment group (resulting in a total of 224 corals). Specimens were housed on raised plastic grids with paired colonies located in adjacent positions at least 10 cm apart, to ensure consistent lighting and surrounding flow for both symbiont types without risk of intercolonial aggression. All colonies were acclimated to their nutritional treatment for three days prior to the start of the experiment so that starved colonies began the trial period with minimum potential benefit of stored nutrition from a previous feeding. During the 60‐day experiment, the starved group received no food while the fed treatment continued to receive offerings of frozen copepod slurry (50 g L^−1^ feeding^−1^). Colonies were carefully observed during these feedings to ensure that each polyp on each colony (a) was given a direct feeding opportunity and (b) demonstrated contraction due to food capture.

### 2.3 Experimental wounding

Colonies were experimentally wounded after the 3‐day treatment acclimation period using a standardized wounding protocol (Burmester, Finnerty, Kaufman, & Rotjan, 2017). A single polyp and the surrounding connective tissue (coenenchyme) were removed from the center of the colony (to control for wound position) using a scalpel before the wound site was cleaned with seawater via Waterpik^®^. Mean wound size was 34.28 mm^2^ (±*SEM* 2.27) with no statistical difference in wound size between groups (ANOVA, *F*_(3,108)_ = 0.9400, *p* = 0.4).

### 2.4 Assessing wound recovery

Colonies were photographed using a Leica M165FC stereomicroscope immediately after wounding and at nine subsequent time points (5, 10, 15, 20, 25, 30, 40, 50, 60 days). The magnification and angle of the photograph were kept constant for each colony across all photographs. These photographs were used to assess wound recovery using two metrics (Burmester et al., 2017). First, each colony was assigned to one of four recovery stages based on a qualitative assessment of the wound site at 60 days post‐wounding: (a) “incomplete occlusion”—the wound remains open or increases in surface area, with bare skeleton still exposed; (b) “full occlusion”—undifferentiated tissue covers a portion or the entirety of the wound site; (c) “tentacle eruption”—tentacle nubs, still incapable of contraction and prey capture, have formed; and (d) “full recovery”—a fully functional polyp capable of feeding has formed at the wound site. Second, we measured the final change in wound surface area (between days 0 and 60). Wound surface area was calculated three times from each photograph using Leica M165FC software, and the resulting mean value was used as the representative wound surface area of that colony for each time point. Proportional final recovery (or tissue loss) was calculated as the difference between the initial wound area (day 0) and final wound area (day 60) divided by initial wound area.

### 2.5 Colony‐wide tissue surface area

Colonies were photographed against a size standard from 6 different angles (top, base, and four sides) at day 0 and day 60. Individual colonies were identified by attaching a honeybee tag (betterbee.com) to the skeletal base using super glue, and the location of this tag was used to ensure consistent directionality to each of the colony's 4 sides. The photographs were manually merged at the end of the experiment to generate a composite image of each colony at day 0 and day 60. To do this, photographs were carefully reviewed for regions of the colony that were photographed from multiple angles, and only the photograph that best represented a given region was retained in the merged images. For the base of the colony, only areas of live tissue growth (and not the entire surface area) were included. The composite area of best represented regions across all photographs was termed the “standard area” for a colony. Both the standard area and area of living tissue within the standard area (“live area”) were determined using ImageJ (NIH). We calculated the proportional live tissue cover (live/standard area) for each colony at day 0 and day 60 and the difference between the initial and final live tissue cover.

### 2.6 Polyp activity

**Figure 2:**
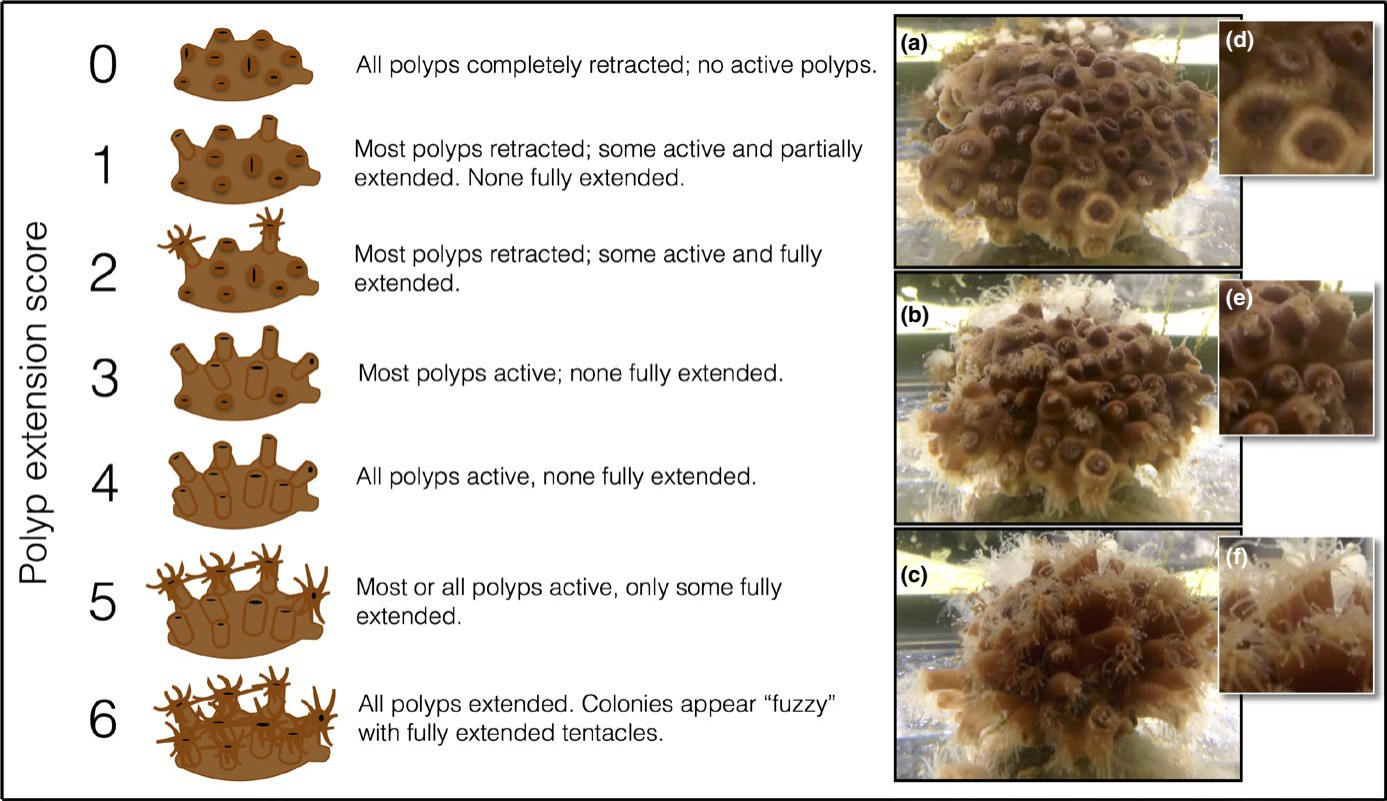
Polyp extension matrix for *Astrangia poculata*. Scores ranged from 0 to 6, with 0 indicating all polyps were fully contracted (a, d) and 6 indicating all polyps are fully extended (c, f). Scores between 1 and 5 represent an intermediate state of polyp extension (b) where different proportions of polyps within a colony are fully contracted (d), active but not fully extended (e), and fully extended (f)

Once per week, polyp activity was scored using a seven‐stage scale (Figure 2): A score of 0 indicated that all polyps were retracted (Figure 2a,d), while a score of 6 indicated the full extension of all polyps within a colony (Figure 2b,e). Scores between 0 and 6 specified intermediate states of increasing polyp extension (Figure 2b,e). A seven‐stage scale was used to enable visually apparent, quantitative distinction between minor, moderate, and major polyp extension, which can be observed as a colony moves from inactive to active (Supporting Information Video S1). Polyp extension values were recorded twice for both fed and starved corals. For fed corals, polyp extension was measured before feeding and 30 min after feeding, to distinguish between basal activity and post‐stimulus activity. For starved corals, polyp extension was measured at the same time points; however, no food was given to this treatment group. Therefore, polyp extension values were recorded twice for both treatment groups, but only the activity of fed colonies was observed in response to a feeding stimulus. Throughout the experiment, all measurements were taken within the same two‐hour range (11:00 hr and 13:00 hr) for each time point to avoid confounds inflicted by diel behavioral cycles.

### 2.7 Quantification of chlorophyll density

Polyp color was used as a proxy for chlorophyll density as previously described (Dimond & Carrington, 2007; Burmester et al., 2017). Colonies were photographed on day 0 and day 60 against red‐green‐blue (RGB) color standards. Photographs were analyzed using custom scripts (Supporting Information S11) on MATLAB R2007b (The MathWorks, Natick, MA), whereby the average of five randomly chosen polyps in a colony was used as the final measurement for each color (red, green, and blue). RGB quantities were condensed to a single principle component (representing overall polyp color) via PCA using a correlation matrix with oblimin rotation. Component loadings were 0.917 for red, 0.721 for green, and 0.870 for blue at Day 0 and 0.898 for red, 0.774 for green, and 0.858 for blue at Day 60. Color PCA values were multiplied by −1 so that increasing color intensity would be associated with increasing PCA values, and the absolute value of the lowest value was added to all color measurements to normalize the data for comparison. As prescribed by Dimond and Carrington (2007), transformed PCA values were converted into chlorophyll density proxies using the equation *y* = 0.044*x* ^2^ + 0.0335*x*(*R* ^2^ = 0.89). Photographs for 60 corals on day 0 and 35 colonies on day 60 were not used in the analysis due to lighting inconsistencies between colonies and the RGB standards, reducing the sample size to 164 specimens at Day 0 and 189 specimens at Day 60.

### 2.8 Photosynthetic efficiency

Photosynthetic efficiency (*F*_v_/*F*_m_) was measured for three randomly selected polyps in each colony in weeks 0, 2, 4, 6, and 8 using a Walz Junior‐PAM pulse‐amplitude modulated fluorescence meter as described in DeFilippo, Burmester, Kaufman, and Rotjan (2016) and Burmester et al. (2017). Briefly, after a thirty‐minute acclimation to darkness, minimal fluorescence (*F*_0_) was measured by exposing polyps to 6 s of far‐red illumination while dark adapted; subsequently, maximal fluorescence (*F*_m_) was determined after exposing polyps to a 0.6 s saturating pulse of 10,000 μmol m^−2 ^s^−1^. Maximum quantum yield (*F*_v_/*F*_m_, unitless) represents the change between maximal and minimal fluorescence over the maximal fluorescence (Suggett et al. (2010)). The resulting *F*_v_/*F*_m _values were averaged for each of the three polyps measured in a given colony at each time point to obtain a single representative value.

### 2.9 Statistical analysis

All statistical analyses were performed using stepwise generalized linear and logistic mixed models (GLMMs) on the lme4 (Bates, Maechler, Bolker, & Walker, 2015) and nlme (Pinheiro, Bates, DebRoy, & Sarkar, 2017) packages in R (R Core Team, 2013). Model selection was based on Akaike's information criterion (AIC) scores, where the decision to include a new fixed‐effect variable or accept one iteration of a model over another required a reduction in AIC of at least 2 (Burnham & Anderson, 2002). The simpler model was always chosen in the case of equal models (Burnham & Anderson, 2002). Additionally, linear models were compared using maximum likelihood tests. For logistic models, odds ratios were calculated using exponentiated estimates. In order to control for the potential impacts of pseudo‐replication that could result from housing multiple colony pairs in the same tank, we used tank as a random effect in all statistical analyses.

Healing initiation (measured as the proportion of colonies in healing stages 2–4 at the end of the study) and healing success (measured as the proportion of colonies that achieved stage 4 at the end of the study, i.e., regenerated fully functional polyps) were tested using Laplace‐approximated logistic GLMMs. The proportional change in wound surface area and total colony surface area were analyzed using restricted maximum likelihood (REML)‐fitted linear GLMMs. In order to test for the impacts of the treatment‐dependent variables as well as independent variables such as lighting and morphological features of the colony and the wound itself, we used a stepwise analysis using nutritional state, symbiont state, mean photosynthetically active radiation (PAR), initial mass, and initial wound size as fixed‐effect variables for wound recovery models. For total colony surface area: Nutritional state, symbiont state, initial mass, PAR, and wounding treatment were used as variables.

Chlorophyll density and polyp extension were analyzed using REML‐fitted linear GLMMs over time (week) with PAR, wounding treatment, nutritional state, initial mass, and symbiotic state as additional variables. In order to control for repeated measurements made on the same individuals over time, colony identity was nested within tank as a random effect. Photosynthetic efficiency was initially tested similarly; however, since time bore no statistically significant effect, a mean maximum quantum yield was calculated over time for each individual colony. Mean photosynthetic efficiency was analyzed using a REML‐fitted linear GLMM using PAR, symbiont state, and nutritional state as fixed‐effect variables.

## 3 RESULTS

### 3.1 Assessing wound recovery

**Figure 3:**
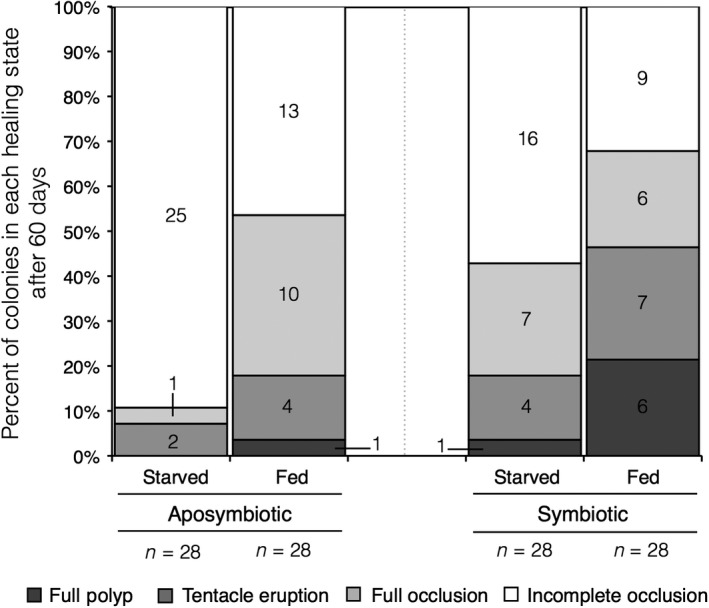
Proportion of colonies in landmark recovery stages (full polyp, tentacle nubs, undifferentiated tissue, or no healing) after 60 days. Bars in all shades of gray collectively represent healing initiation, while bars in dark gray represent developmental healing success. Numbers in bars signify total number of colonies in each stage

Both nutritional state and symbiont state played a significant role on healing initiation. After accounting for tank grouping using random effects, symbiotic colonies (31 colonies or 55.3%) were 3.021 times more likely than aposymbiotic colonies (18 colonies or 32.1%) to reach any of the three landmark developmental stages (undifferentiated tissue, tentacle nubs, or full polyps; Figure 3, Supporting Information Table S1). Likewise, nutritional state had a strong impact on healing success: Fed colonies (34 colonies or 60.7%) were 4.692 times more successful than starved colonies (15 colonies or 26.8%; Figure 3). Both symbiont state and nutritional state (but not their interaction, PAR, initial mass, and initial wound size) were significant predictors of healing initiation (AIC 141.1, symbiont state: *p* < 0.01, nutritional state: *p* < 0.001, Supporting Information Table S1). Only symbiont state significantly impacted healing success (the formation of fully functional tentacles) according to GLMM analysis (AIC 58.1, *p* < 0.0001, Supporting Information Table S2). In order to adjust for the small sampling of aposymbiotic colonies with full polyp development (1/56 colonies, Figure 3), a second GLMM was performed on only the subset of symbiotic colonies. However, this model did not find nutritional state to be significant (AIC 43.7, *p* = 0.07, Supporting Information Table S3), which could potentially derive from a lack of statistical power. Accounting for the tank random effect, symbiotic (7/56) colonies were 8.013 times more likely than aposymbiotic colonies (1/56) to successfully complete the developmental recovery process (Figure 3).

**Figure 4:**
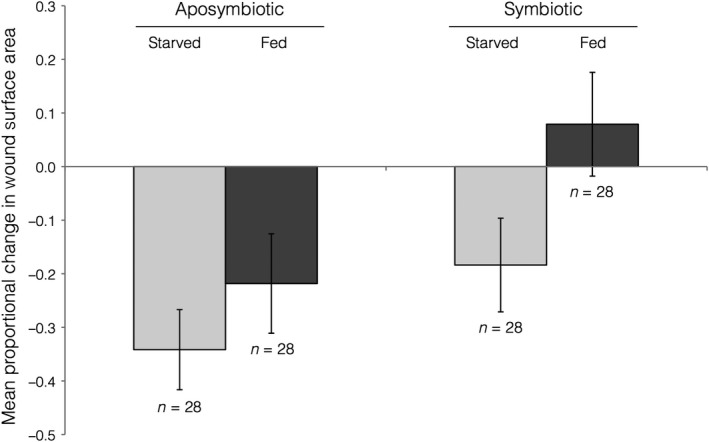
Mean proportional change in wound surface area 60 days after lesions were induced. Error bars signify standard error

**Figure 5:**
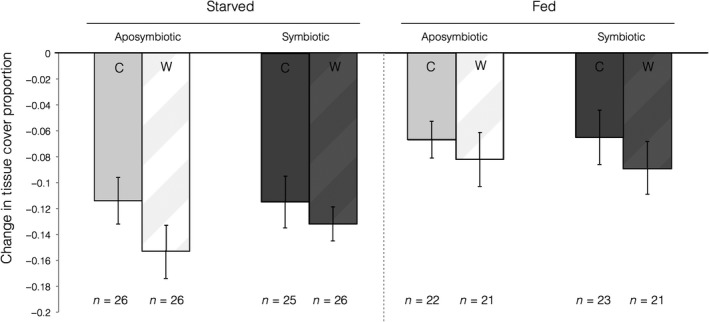
Mean proportional change in total colony tissue cover proportion after 60 days for control (C) and wounded (W) fed and starved colonies of different symbiont states (aposymbiotic, symbiotic). Error bars signify standard error

Symbiont state, but not any other fixed effects (nutritional state, PAR, initial mass, and initial wound size or their interactions), was a significant predictor of proportional wound surface area recovery (AIC 152.4, *p* < 0.01, Supporting Information Table S4). On average, only the symbiotic fed treatment group (mean ± *SEM*: 0.079 ± 0.096 proportional units) exhibited wound recovery via a reduction in wound size (shown here as a proportional increase in live tissue surface area; Figure 5). Wound size increased over time for all aposymbiotic colonies (mean ± *SEM*: starved, −0.3416 ± 0.0748; fed, −0.2183 ± 0.0928) and starved, symbiotic colonies (−0.1836 ± 0.0874; Figure 4). While no group demonstrated full recovery across all colonies, the greatest proportion (16/28 or 57.14%) of colonies with wound closure was for the symbiotic, fed treatment group. In the remaining groups, less than half of the wounded colonies exhibited live tissue recovery at the wound site: 9/28 (32.14%) for aposymbiotic fed, 8/28 (28.57%) for symbiotic starved, and 4/28 (14.29%) for aposymbiotic starved corals.

### 3.2 Colony‐wide tissue surface area

Overall, starved colonies (mean ± *SEM*, −0.1253 +/0.0126) experienced a greater (nearly double) decline in proportional colony surface area than did fed colonies (mean ± *SEM*, −0.0728 ± 0.0138; Figure 5). According to the most parsimonious model, there was no significant impact of wounding treatment, symbiotic state, initial mass, or PAR; however, nutritional state did play a slight but significant role in predicting changes in total colony surface area (AIC −354.9, *p* = 0.0283), Supporting Information Table S5). Additionally, fed treatment groups experienced a higher proportion of colonies with increased total colony live tissue surface area (8/44 or 18.18% for symbiotic colonies; 7/43 or 16.28% for aposymbiotic colonies) than did starved colonies (3/51 or 5.88% for symbiotic colonies; 5/52 or 9.61% for aposymbiotic colonies).

### 3.3 Polyp activity

**Figure 6:**
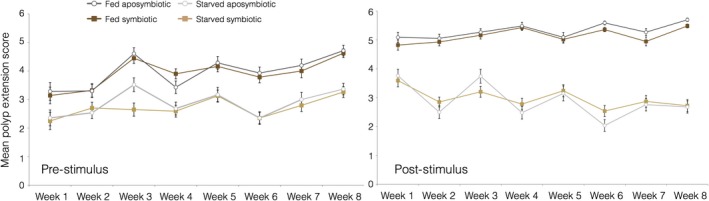
Mean polyp extension scores across a 60 day (8 weeks) period both before (pre‐) and after (post‐) a food stimulus had been supplied to the fed treatment group. Starved (symbiotic and aposymbiotic) colonies were provided no food stimulus

Fed colonies consistently exhibited higher polyp extension scores than starved corals both before and after a stimulus was provided to fed colonies (Figure 6). For both pre‐ and post‐stimulus models, wounding treatment, symbiotic state, PAR, and initial mass had no significant impact on polyp extension (Supporting Information Table S6: AIC 5874.1; Supporting Information Table S7, AIC 5611.4). The best models for both stimulus regimes selected nutritional state, time, and the interaction of time and nutritional state as significant predictive fixed effects (*p* < 0.05, Supporting Information Tables S6)and S7). In order to test for the impact of applying a food‐related stimulus, an additional REML‐fitted GLMM was performed on the subset of fed corals (pre‐ and post‐stimulus). This model found both time and applied stimulus to be significant predictors of polyp extension (AIC 5642.9, *p* < 0.05, Supporting Information Table S8), whereby polyp extension varied over time but was consistently higher than pre‐stimulus colonies after food was supplied.

### 3.4 Quantification of chlorophyll density

**Figure 7:**
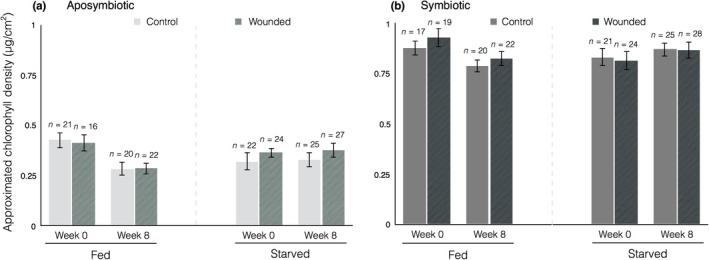
Mean chlorophyll density as determined by RGB color values. Error bars represent standard error

Throughout the experiment, regardless of symbiotic state, there was no significant difference between fed and starved colonies in chlorophyll density (Figure 7). Symbiotic colonies had greater approximated chlorophyll density (ACD) (mean ± *SEM*, 0.837 ± 0.016 µg/cm) than aposymbiotic colonies (mean ± *SEM*, 0.347 ± 0.013 µg/cm) at all time points and under all experimental conditions (Figure 7). The most parsimonious model selected three significant fixed effects (symbiotic state, time, and the interaction of nutritional state and time) as well as one non‐significant predictor—nutritional state (AIC −239.93, *p* < 0.05, Supporting Information Table S9). This analysis is congruous with a variation in ACD between the initial (Day 0) and final (Day 60) measurements and a consistent decline in ACD over time among fed corals. The strongest predictor (by estimate), however, was symbiotic state (Supporting Information Table S9).

### 3.5 Photosynthetic efficiency

**Figure 8:**
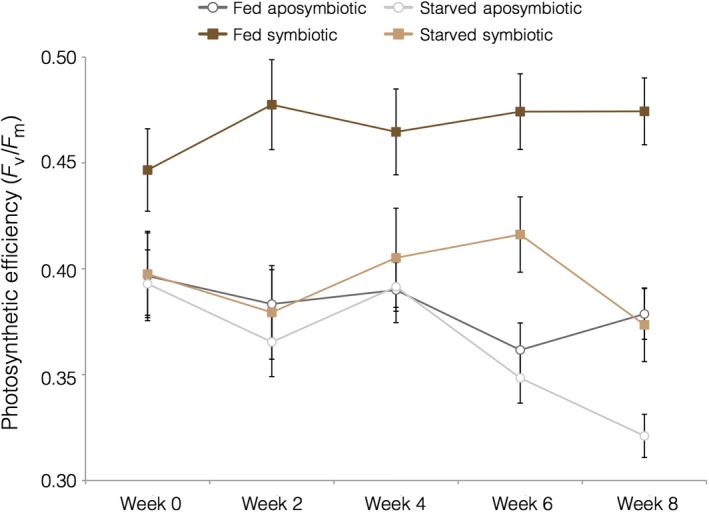
Mean maximum quantum yield (*F*_v_/*F*_m_) across a 60‐day (8 weeks) period. Error bars signify standard error

Because time was not found to be a significant predictor of photosynthetic efficiency, we analyzed maximum quantum yield (*F*_v_/*F*_m_, unitless) averaged across all five time points. Symbiotic, fed colonies exhibited significantly greater photosynthetic efficiency (mean ± *SEM*, 0.394 ± 0.011) than all other groups at all time points (Figure 8). Mean maximum quantum yield was similar among starved, symbiotic colonies (mean ± *SEM*, 0.394 ± 0.011) and aposymbiotic colonies (mean ± *SEM*; fed: 0.382 ± 0.009, starved: 0.364 ± 0.007; Figure 8). Consistent with these results, the model with best support found symbiont state (*p* < 0.0001) and the interaction between symbiotic state and nutritional state (particularly among symbiotic and not aposymbiotic colonies, *p* < 0.0001) to be the most significant predictors of photosynthetic efficiency (AIC 486.8, Supporting Information Table S10).

## 4 DISCUSSION

Our findings highlight some of the dynamic pathways through which coral colonies might obtain, distribute, and utilize energetic resources during the process of recovering from physical abrasion. This study suggests that autotrophy plays an important role in wound recovery and that there may be an important interplay and feedback (both positive and negative) between autotrophy and heterotrophy. As previously found in *A. poculata*, symbiotic state had a significant role on healing initiation and success as well as proportional surface area recovery to wounds (Burmester et al., 2017; DeFilippo et al., 2016). However, symbiont state alone was not enough to maximize healing potential. Starved‐symbiotic and fed aposymbiotic healed comparably, while there was an additive negative feedback between starved aposymbiotic corals (no nutrition from either source; little/no healing), and an additive positive feedback between fed symbiotic corals (nutrition from both sources; highest healing ability) (Figures 3 and 4).

While nutritional state impacted healing initiation, it had no statistical effect on healing success or surface area recovery, presumably because there was some autotrophic compensation. On the other hand, only nutritional treatment (and not symbiont state) appeared to play a role in total colony tissue maintenance. These findings suggest that energy might not be regulated or distributed uniformly across levels of body organization, which is to be expected in a colonial organism that can translocate resources. This is consistent with other studies, where branching growth tips of *Stylophora pistillata *had significantly less ^14^C products than fragments from below branch tips (Rinkevich & Loya, 1983). Additionally, both symbiotic state and lesion induction can alter the quantity and directionality of carbon translocation across a coral colony (Fine et al., 2002; Oren et al., 1997). In *O. patagonica*, preferential translocation to recovering tissue proceeded from a distance of 4–5 cm, but this phenomenon does not occur in colonies that were fully or partially (30%–80%) bleached (Fine et al., 2002). The pace and completion of wound recovery are subject to the impacts of several intrinsic and extrinsic factors (such as colony size, wound size, wound location, temperature, disease state, sedimentation [as reviewed by Henry & Hart, 2005 and for example: Van Veghel & Bak, 1994, Meesters, Noordeloos, & Bak, 1994, Meesters, Wesseling, & Bak 1996, Meesters, Pauchli, & Bak, 1997, Nagelkerken & Bak, 1998, Nagelkerken, Meesters, & Bak, 1999, Kramarsky‐Winter & Loya, 2000, Rotjan & Lewis, 2005, Edmunds, 2009, Denis et al., 2011, Cameron & Edmunds, 2014]), which also have the potential to interact with energy sourcing and nutritional state. The type of damage inflicted may also play a role in how energy is regulated or redirected to recovery and other biological processes (DeFilippo et al., 2016). Dislodged colonies of *Pocillopora damicornis* with edge damage experience a decrease in overall energy allocation, resulting in higher mortality rates and decreased growth and reproduction (Ward, 1995). Meanwhile, fragmentation bears no significant impact on growth and mortality, but results in higher overall energy allocation and increased reproduction (Ward, 1995). Therefore, it is likely that tissue maintenance and damage are regulated differently for small‐scale local wounds (e.g., the single polyp removal demonstrated in this study) and across a coral's total colony tissue cover (e.g., DeFilippo et al., 2016), potentially due to underlying compartmentalized nutritional gradients across an energetically integrated colony (Conlan, Humphrey, Severati, & Francis, 2018). Interestingly, our results indicate that symbiont state is more important to the regulation of tissue surface area at the wound level while overall maintenance of total colony tissue cover is more greatly impacted by the presence or absence of prey items. Therefore, there could be an added cost to lesion recovery during and after bleaching events that may not be fully supplemented via heterotrophy. This is consistent with tropical corals that rely more heavily on autotrophy; for example, wounded *Orbicella *colonies recovered more slowly from bleaching compared to intact colonies (Rotjan et al., 2006). Additionally, this added cost may be compounded by the influence of other external disturbances to wound recovery, such as ocean acidification (Edmunds & Yarid, 2017) or elevated sea surface temperatures (Bonesso, Leggat, & Ainsworth, 2017). Since recent evidence also suggests that physiological integration (i.e., high integration) may increase risk of bleaching stress (Swain et al., 2018), understanding how corals utilize, store, and distribute energy from multiple nutritional sources may prove invaluable to conservation efforts.

Both the availability (stimulus) of prey items and the history of heterotrophic opportunity significantly influenced polyp foraging behavior. Fed colonies maintained a higher degree of polyp expansion than unfed colonies at all time points, and the introduction of food particles induced even greater expansion. In tropical, obligate symbiotic scleractinians, symbiotic photosynthetic energy resources have been shown to influence heterotrophic activity. Colonies of *Pocillopora damicornis* maintained under dark conditions for 2 weeks ingested less *Artemia* nauplii than those in lighted conditions, suggesting a dependence on energy from photosynthesis to meet the metabolic needs required for sustainable foraging behavior (Clayton & Lasker, 1982). In the present study, there was no observed statistical difference in foraging activity between symbiotic and aposymbiotic colonies. These results are similar to those found for other facultatively symbiotic corals. Piniak (2002) found that prey capture efficiency varied by prey type and flow rate, but observed no difference between (fed) symbiotic and aposymbiotic colonies of *Oculina arbuscula*. Coral colonies may also forage advantageously regardless of photosynthetic activity, as even obligate, tropical corals have been shown to seek heterotrophic nutrition even if metabolic carbon requirements are met via autotrophy (Ferrier‐Pagès, Allemand, Gattuso, & Jaubert, 1998; Grottoli et al, 2006). Additionally, the regular availability of heterotrophic food sources increased foraging activity in fed colonies both with and without a food stimulus. Therefore, colonies with stable nutritional inputs are better able to maintain a fuller, long‐term foraging effort, allowing them to not only respond to a food stimulus, but to also survey their environment. This suggests a heterotrophic, rather than autotrophic, mechanism for inducing appropriate behavior to meet metabolic demands in temperate, facultatively symbiotic corals.

In this study, while the photosynthetic efficiency (maximum quantum yield) of fed symbiotic colonies was significantly higher than that of all aposymbiotic (fed and starved) colonies, there was no difference between aposymbiotic colonies and starved‐symbiotic colonies. This phenomenon does not appear to derive from a loss of chlorophyll, which suggests an energetic cost to symbiont photosynthesis that must be fulfilled via host heterotrophic means. In fact, zooxanthellae have been documented to exhibit heterotrophic behavior inducing a parasitic metabolic burden on the facultatively symbiotic anemone *Aiptasia pulchella* (Steen 1986; Baker, Freeman, Wong, Fogel, & Knowlton, 2018). Previous studies have documented an enhancement to photosynthesis in temperate corals after feeding (Jacques & Pilson, 1980). Similarly, rates of photosynthesis increased (2–10×) after the introduction of heterotrophic food sources to *Stylopora pistilla* (Houlbrèque et al., 2003). The decline in photosynthetic efficiency for starved, symbiotic colonies could also potentially be attributed to their higher rates of polyp contraction. *A. poculata* go through a winter quiescence phase, when polyps enter a state of metabolic dormancy (Jacques et al., 1983) and tentacles no longer elicit a tactile feeding response (Grace, 2017). Quiescence corresponds with wintertime food scarcity in New England due to relatively oligotrophic waters compared to summer nutrient conditions and corresponding plankton blooms (Grace, 2017). During quiescence, *A. poculata* colonies in New England experience a decline in photosynthetic efficiency and in ACD (Dimond & Carrington, 2007). Although the cold wintertime temperatures have assumed to be a driver of quiescence behavior, the polyp behavior and photosynthetic efficiency of starved corals in our experiment suggest that quiescence may instead have a nutritional cue, since ambient temperatures (18°C) were maintained throughout the experiment. Photoperiod and/or angle of incidence may also play a role, as Fabricius and Klumpp (1995) found reduced photosynthetic productivity and increased required levels of irradiance to achieve photosynthetic compensation and saturation in contracted large‐polyped soft corals. Though again, PAR was maintained throughout the 60 days of this experiment.

The dynamic relationship between *Astrangia poculata* and *Symbiodinium psygmophilum* is well‐documented, with both symbiotic states characterized across its range (Dimond et al., 2013), and the potential for state‐switching under experimental conditions (Dimond & Carrington, 2007). The aposymbiotic state is common in nature (Grace, 2004) despite relevant losses in recovery ability (Burmester et al., 2017; DeFilippo et al., 2016) as well as resilience to stress (Holcomb, Cohen, & McCorkle, 2012; Holcomb, McCorkle, & Cohen, 2010). It has been hypothesized that the persistence of the aposymbiotic life history may be due, in part, to the relative reduction in polyp loss under cold temperatures during winter quiescence (Dimond et al, 2013). Despite its thermal tolerance and resilience to chronic cold exposure (Thornhill, Kemp, Bruns, Fitt, & Schmidt, 2008), *S. psygmophilum* experiences a rapid decline and cessation in maximum quantum yield at winter temperatures. Combined with metabolic dormancy and a lack of feeding response, the demonstrated decline in photosynthetic efficiency in the absence of heterotrophy in implied energetic cost of these symbionts to the host coral could explain the reduced polyp loss (and correspondingly higher biomass compared to symbiotic corals) of aposymbiotic colonies under overwinter conditions.

While feeding behavior was ensured among all polyps for each colony, this study did not specifically determine the quantity of food consumed nor the amount of carbon incorporated. Likewise, while we recorded light availability (PAR) and photosynthetic efficiency (*F*_v_/*F*_m_), neither of these measurements provide accurate insight into photosynthetic carbon production for this coral. As such, it would be difficult to infer how specific pathways might be impacted by differences in symbiont state and experimental feeding treatments on the cellular level. Additionally, these results represent the nutritional dynamics of a single, facultatively symbiotic species that may not be directly applicable to tropical coral species with higher dependencies on autotrophic pathways. However, the results of this study demonstrate significant and predictable morphological and stress‐tolerant responses that influence key life history strategies in temperate corals and broadly highlight the importance in understanding the complexity of energy sourcing when establishing energy budgets for maintaining organismal health.

## AUTHOR CONTRIBUTIONS

EMB, RDR, JRF, and LK conceived the ideas and designed methodology. EMB, NFL, and ABP collected the data. EMB analyzed the data and generated figures. All authors contributed critically to the drafts and gave final approval for publication.

## DATA ACCESSIBILITY

Data are publicly available via the Dryad Digital Repository (https://doi.org/10.5061/dryad.tt7p900).
