# Supplementary material for: The impact of autotrophic versus heterotrophic nutritional pathways on colony health and wound recovery in corals
Source: Ecol Evol. 2018 Oct 18;8(22):10805–16. doi: 10.1002/ece3.4531 (PMC6262932; doi:10.1002/ece3.4531)
Supplement: Supplementary file 1 [file ECE3-8-10805-s001.docx]

**Supplemental Tables & Information**

Table S1. Laplace approximated generalized mixed logistic regression for healing initiation (AIC = 141.1). For fixed effects, ** indicates significance at α< 0.001, * indicates significance at α= 0.05.

| **Effect** | **Estimate** | **Standard Error** | **Z-Value** | **P-Value** |
| --- | --- | --- | --- | --- |
| Intercept | -0.08403 | 0.33976 | -0.247 | 0.8047 |
| Symbiont State (S) | 1.10561 | 0.42719 | 2.588 | 0.0096* |
| Nutritional State (F) | 1.54582 | 0.42838 | -3.609 | 0.0003** |
| ^#^Rooted under Aposymbiotic (A) and Starved (St) conditions | | | | |

Table S2. Laplace approximated generalized mixed logistic regression for healing success (polyp formation) (AIC = 58.1). For fixed effects, ** indicates significance at α< 0.001, * indicates significance at α= 0.05.

| **Effect** | **Estimate** | **Standard Error** | **Z-Value** | **P-Value** |
| --- | --- | --- | --- | --- |
| Intercept | -4.099128 | 0.009418 | -435.2 | <0.0001** |
| Symbiont State (S) | 2.081012 | 0.009417 | 221.0 | <0.0001** |

Table S3. Laplace approximated generalized mixed logistic regression for healing success (polyp formation) for symbiotic corals only (AIC = 43.7). For fixed effects, ** indicates significance at α< 0.001, * indicates significance at α= 0.05, ^+^ indicates significance at α= 0.1.

| **Effect** | **Estimate** | **Standard Error** | **Z-Value** | **P-Value** |
| --- | --- | --- | --- | --- |
| Intercept | -1.2993 | 0.4606 | -2.821 | 0.00479* |
| Nutritional State (F) | 1.9966 | 1.1177 | -1.786 | 0.07474^+^ |

Table S4. REML-fitted linear mixed model for wound surface area (AIC 152.4). For fixed effects, ** indicates significance at α< 0.001, * indicates significance at α= 0.05.

| **Effect** | **Estimate** | **Standard Error** | **DF** | **T-Value** | **P-Value** |
| --- | --- | --- | --- | --- | --- |
| Intercept | -0.2787 | 0.0956 | 103 | -2.9142 | 0.0044* |
| Symbiont State (S) | 0.2277 | 0.0810 | 103 | 2.8122 | 0.0059* |

Table S5. REML-fitted linear mixed model for total colony surface area (AIC -354.9). For fixed effects, ** indicates significance at α< 0.001, * indicates significance at α= 0.05.

| **Effect** | **Estimate** | **Standard Error** | **DF** | **T-Value** | **P-Value** |
| --- | --- | --- | --- | --- | --- |
| Intercept | -0.0755 | 0.0126 | 182 | -6.0034 | <0.0001** |
| Nutritional State (F) | 0.0497 | 0.0173 | 6 | -2.8729 | 0.0283* |

Table S6. REML-fitted generalized linear mixed model for pre-stimulus mean polyp extension scores (AIC 5874.1). Significance (*) assumed from t-values (DF= 7) at α= 0.05.

| **Effect** | **Estimate** | **Standard Error** | **T-Value** |
| --- | --- | --- | --- |
| Intercept | 4.9416 | 0.1061 | 46.59* |
| Nutritional State (St) | -1.5248 | -0.1517 | -10.05* |
| Time | 0.0643 | 0.0160 | 4.02* |
| Nutritional State (St): Time | -0.1718 | 0.0231 | -7.45* |

Table S7. REML-fitted generalized linear mixed model for post-stimulus mean polyp extension scores (AIC 5611.4). Significance (*) assumed from t-values (DF= 7) at α= 0.05.

| **Effect** | **Estimate** | **Standard Error** | **T-Value** |
| --- | --- | --- | --- |
| Intercept | 3.4621 | 0.1404 | 24.658* |
| Nutritional State (St) | -0.8249 | 0.2001 | -4.123* |
| Week | 0.1200 | 0.0210 | 5.727* |
| Nutritional State (St): Week | -0.0744 | 0.0300 | -2.476* |

Table S8. REML-fitted generalized linear mixed model for mean polyp extension scores (pre- and post-stimulus) for fed corals (AIC 5642.9). Significance (*) assumed from t-values (DF= 5) at α= 0.05.

| **Effect** | **Estimate** | **Standard Error** | **T-Value** |
| --- | --- | --- | --- |
| Intercept | 4.8161 | 0.0879 | 54.82* |
| Stimulus (Pre) | -1.2406 | 0.0572 | -21.68* |
| Time | 0.0934 | 0.0127 | 7.36* |

Table S9. REML-fitted generalized linear mixed model for approximated chlorophyll density (AIC -239.93). Significance (*) assumed from t-values (DF= 8) at α= 0.05.

| **Effect** | **Estimate** | **Standard Error** | **T-Value** |
| --- | --- | --- | --- |
| Intercept | 0.4129 | 0.0223 | 18.494* |
| Symbiotic State (S) | 0.4966 | 0.0202 | 24.601* |
| Nutritional State (St) | -0.0487 | 0.0268 | -1.816 |
| Week | -0.0149 | 0.0031 | -4.856* |
| Nutritional State (St): Week | 0.0108 | 0.0041 | 2.644* |

Table S10. REML-fitted linear mixed model for mean maximum quantum yield (Fv/Fm) (AIC -486.8). For fixed effects, ** indicates significance at α< 0.001, * indicates significance at α= 0.05, ^+^ indicates significance at α= 0.1.

| **Effect** | **Estimate** | **Standard Error** | **DF** | **T-Value** | **P-Value** |
| --- | --- | --- | --- | --- | --- |
| Intercept | 0.3795 | 0.0128 | 211 | 29.7495 | <0.0001** |
| Symbiont State (S) | 0.0846 | 0.0138 | 211 | 6.1153 | <0.0001** |
| Symbiont State (A): Nutritional State (St) | -0.0119 | 0.0170 | 211 | -0.7027 | 0.4830 |
| Symbiont State (S): Nutritional State (St) | -0.0660 | 0.0170 | 211 | -3.8850 | 0.0001** |

Supplementary Information S11. Matlab script for quantifying RGB values from photographs. Script coded by J. Chabot.

%coral.m--collect RGB statistics for coral pictures

clear

close

%read text file containing a) total number of pictures and b) filenames

fdir='c:\matlab6p5\work\pics\';

flist=fopen([fdir 'flist.txt'],'r');

%output file

fout=fopen([fdir 'results.dat'],'w');

%header

fline=['Image CoralR CoralG CoralB RedR RedG RedB GreenR GreenG GreenB BlueR BlueG BlueB "Red" "Green" "Blue" \n'];

fprintf(fout,fline);

%read number of image files to process

fnum=str2num(fgetl(flist));

%warning off MATLAB:divideByZero;

for imageloop=1:fnum

    %open image

    imname=fgetl(flist);

    origpic=imread([fdir imname],'jpeg');

    rawpic=origpic(1:3:end,1:3:end,:);

    %user identifies regions of interest: coral, stripes (red, green blue)

    regcoral=sparse(roipoly(rawpic)); %for coral

    regred=sparse(roipoly(rawpic)); %for red

    reggreen=sparse(roipoly(rawpic)); %for green

    regblue=sparse(roipoly(rawpic)); %for blue

    %get mean and std for each region

    for structloop=1:4

        switch structloop

            case 1

                reg=regcoral;

            case 2

                reg=regred;

            case 3

                reg=reggreen;

            case 4

                reg=regblue;

        end %switch

        %initial guesses for bands

        for colorloop=1:3

            meanlev(structloop,colorloop)=mean(nonzeros(double(rawpic(:,:,colorloop)).*reg));

            stdev(structloop,colorloop)=std(nonzeros(double(rawpic(:,:,colorloop)).*reg));

        end %colorloop

    end %structloop

    dilatemask=strel('disk',2);

    %expand colored bands and get mean and std for each

    for structloop=2:4

        switch structloop

            case 2

                reg=regred;

            case 3

                reg=reggreen;

            case 4

                reg=regblue;

        end %switch

        for colorloop=1:3

            test(:,:,colorloop)=(rawpic(:,:,colorloop)>=(meanlev(structloop,colorloop)-2*stdev(structloop,colorloop)))&...

                (rawpic(:,:,colorloop)<=(meanlev(structloop,colorloop)+2*stdev(structloop,colorloop)));

        end %colorloop

        colorreg=sparse(false(size(rawpic)));

        %region is original area selected + all pixels within 2SD of all three

        %colors

        colorreg=or(reg,(test(:,:,1) & test(:,:,2) & test(:,:,3)));

        %keep pixels connected to original area

        colorreg=imreconstruct(full(reg),colorreg);

        %fill holes in retained region

        colorreg=imfill(colorreg,'holes');

        %kill anything less than ten pixels wide

        colorreg=imdilate(imerode(colorreg,dilatemask),dilatemask);

        %savereg(:,:,structloop-1)=colorreg; %if we want to store the region

        %to verify that it is correct

        for colorloop=1:3

            meanlev(structloop,colorloop)=mean(nonzeros(colorreg.*double(rawpic(:,:,colorloop))));

            stdev(structloop,colorloop)=std(nonzeros(colorreg.*double(rawpic(:,:,colorloop))));

        end %colorloop

    end %structloop

    meanRGB=(meanlev(2:4,:))';

    meancoral=(meanlev(1,:))';

    components=inv(meanRGB)*meancoral;

    fline=[imname ' ' num2str(meanlev(1,:)) ' ' num2str(meanlev(2,:)) ' ' num2str(meanlev(3,:)) ' ' num2str(meanlev(4,:)) ' ' num2str(components') '\n'];

    fprintf(fout,fline);

end %imageloop

fclose(flist);

fclose(fout);

Supplementary Video S1. Polyp extension behavior in *A. poculata.* Video taken by S. Pelletier.
